# Combining Mendelian Randomization and Experimental Validation to Reveal the Causal Relationship Between Hallux Valgus and Serum Metabolites and to Identify Their Therapeutic Targets and Relevant Components

**DOI:** 10.1002/fsn3.72143

**Published:** 2026-07-23

**Authors:** Xin Li, Xuefeng Luo, Liqi Ng, Peng Lu, Wei Huang, Min Huang, Songchuan Su, Yu Zhou

**Affiliations:** ^1^ Pharmacy Department Orthopedic Hospital, Chongqing University of Chinese Medicine Chongqing China; ^2^ Orthopedic Laboratory of Chongqing Medical University Chongqing China; ^3^ Department of Orthopedics, Orthopedic Laboratory of Chongqing Medical University The First Affiliated Hospital of Chongqing Medical University Chongqing China; ^4^ Institute of Orthopaedic and Musculoskeletal Science University College London, Royal National Orthopaedic Hospital Stanmore, London UK; ^5^ Foot and Ankle Surgery Department Orthopedic Hospital, Chongqing University of Chinese Medicine Chongqing China; ^6^ Chongqing Geleshan Community Health Centre Chongqing China; ^7^ Postdoctoral Research Workstation Orthopedic Hospital, Chongqing University of Chinese Medicine Chongqing China; ^8^ Department of Spine Surgery Orthopedic Hospital, Chongqing University of Chinese Medicine Chongqing China; ^9^ Laboratory of Chinese Medicine Orthopedics and Traumatology Chongqing University of Chinese Medicine Chongqing China

**Keywords:** cysteine, hallux valgus, Mendelian randomization, natural marine compounds, serum metabolites

## Abstract

Hallux valgus (HV) is a common foot deformity influenced by genetic, pathological, and traumatic factors, yet its relationship with serum metabolites (SMs) remains unclear. This study employed two‐sample Mendelian randomization (SMR) to investigate causal associations between 1400 SMs and HV, identifying Cysteine and Glycochenodeoxycholate 3‐sulfate as negatively correlated with HV occurrence, with no significant reverse causal effect from HV on their levels. Focusing on Cysteine, 548 target genes were predicted from four databases (DrugBank, SuperPred, Genecards, and SwissTargetPrediction) and 793 HV‐related genes were retrieved from Genecards, yielding 47 overlapping genes. KEGG enrichment analysis revealed that these intersecting genes were involved in pathways such as cellular senescence and the AGE‐RAGE signaling pathway in diabetic complications. Among them, fibroblast growth factor receptor 2 (FGFR2) showed a positive causal relationship with Cysteine and a negative causal relationship with HV, which was validated in patient samples showing significantly lower serum Cysteine levels and bone tissue FGFR2 expression in the HV group compared to non‐HV controls (*p* < 0.001). Additionally, Isogranulatimide was identified as a marine‐derived active compound targeting FGFR2, with molecular docking and dynamic simulations confirming favorable binding activity. These findings suggest that Cysteine may play a protective role in the development and progression of HV, with FGFR2 serving as a key mediating target that could reduce HV risk both indirectly through elevated Cysteine levels and directly through its own regulation, offering valuable reference for targeted diagnosis and therapy in clinical practice.

## Graphical abstract

This study employed a two‐sample Mendelian randomization approach to investigate the causal relationship between 1400 serum metabolites and hallux valgus (HV). Cysteine was identified as a key metabolite with a significant negative causal association with HV. Through multi‐database target prediction and intersecting gene analysis, FGFR2 was pinpointed as the critical intersection target, exhibiting a positive causal relationship with Cysteine and a negative causal relationship with HV. Clinical validation in patient serum and bone tissue samples confirmed significantly reduced Cysteine and FGFR2 levels in HV patients. Furthermore, the marine natural compound Isogranulatimide was predicted and validated as a potential FGFR2‐targeting therapeutic agent through molecular docking and molecular dynamics simulations. Collectively, our findings establish the Cysteine‐FGFR2 axis as a novel pathogenic mechanism in HV, providing a theoretical foundation for targeted diagnosis and treatment strategies.

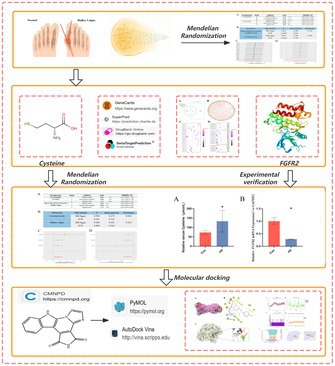

**Abbreviations**
CMNPD: Comprehensive Marine Natural Products Database
Elisa: Enzyme‐linked immunosorbent assay
FDR: False Discovery Rate
FEL: The free energy landscape
FGFs: Fibroblast growth factors
HV: Hallux valgus
IVs: Instrumental variables
IVW: Inverse variance weighted
LD: Link‐age disequilibrium
ME: MREgger
MR‐PRESSO: MR pleiotropy residual sumand outlier
NAC: N‐acetylcysteine
qRT‐PCR: Quantitative reverse transcription‐polymerase chain reaction
Rg: Radius of Gyration
RMSD: Root Mean Square Deviation
RMSF: Root Mean Square Fluctuation
SM: Simple mode
SMR: Two‐sample Mendelian randomization
SMs: Serum metabolites
SNPs: Single nucleotide polymorphisms
WM: Weighted median
WM: Weighted mode

## 1 Introduction

Hallux valgus (HV) is a common foot deformity that presents as a lateral deviation of the first metatarsophalangeal joint. Studies have shown that the global prevalence of HV is 19% or higher, with a particularly high prevalence in Asia and Oceania (Cai et al. 2023; Lu et al. 2025). HV can affect people of all ages, from children as young as 10 years of age and is more prevalent in people over 60 years of age, with a significantly higher prevalence in women than in men (Knörr et al. 2022). The pathogenesis of HV is not yet clear, but valgus deformity is usually caused by a combination of factors, including genetic factors, muscle imbalance, abnormal joint structure, etc. Clinicians are more likely to believe that it is related to the individual's shoe‐wearing habits or other biomechanical factors (Jia et al. 2021; Hsu et al. 2015).

Serum metabolites (SMs) are small‐molecule organic compounds in the blood that reflect the physiological and pathological states of the body. The composition and levels of SMs are influenced by a variety of factors, including genetics, the environment, lifestyle, and health status, and alterations in SMs are mainly related to the metabolism of energy, lipids, and amino acids (Yu et al. 2022b). The study of SMs can provide important information for the diagnosis, prevention, and treatment of diseases (von Rundstedt et al. 2016).

Currently, there is a lack of clinical research on the association between SMs and the onset and progression of HV. In recent years, MR has been widely used in various fields as a method capable of inferring potential causality by using single nucleotide polymorphisms (SNPs) as instrumental variables (IVs) to assess causality between exposure factors and outcomes. Therefore, this study investigated the causal relationship between 1400 SMs and HV by SMR analysis. In addition, the intersecting key targets between key SMs and HV were clarified, and the causal relationship between them was verified in reverse. Finally, the screening of natural marine compounds related to the key targets provides some basis for targeted treatment of HV. In conclusion, this study provides important insights into the causal relationship between SMs, especially Cysteine, and HV, and provides certain theoretical references for the prevention, diagnosis, and treatment of HV patients.

## 2 Materials and Methods

### 2.1 Data Sources and Methods of Analysis

This Mendelian randomization study was conducted and reported in accordance with the STROBE‐MR guidelines, with detailed items provided in Table S1 (Skrivankova et al. 2021). MR methods were used to examine the causal relationship between 1400 SMs and HV in a European population. Based on the search of the GWAS database (http://gwas.mrcieu.ac.uk), the “finn‐b‐M13_HALLUXVALGUS” dataset was used as the source of the GWAS data on HV, which included 155,757 individuals of European origin diagnosed with HV (ncase = 8536, ncontrol = 147,221, 165, SNPs = 147,221, 165, SNPs = 147,221, 165, SNPs = 147,221) individuals diagnosed with HV (ncase = 8536, ncontrol = 147,221, 165, SNPs = 16,380,195). In addition, the GWAS dataset of 1400 SMs was obtained from Chen et al. (2023). The study involved 8299 individuals and covered the most comprehensive range of human SMs, totalling 1091 SMs and 309 metabolite ratios (https://www.ebi.ac.uk/gwas/, GCST90199621‐GCST90201020). In this study, Random‐effects inverse variance weighted (IVW), Weighted median (WM), MREgger (ME), Simple mode (SM) and Weighted mode (WM) were used as the analytical methods, where IVW was used to assess the relationship between exposure factors and outcomes; we applied Cochran's Q test (*p* < 0.05) to estimate residual heterogeneity for the IVW model and the MR‐Egger intercept test (*p* < 0.05) to indicate potential pleiotropy on causal estimates (Cohen et al. 2015; Burgess and Thompson 2017). A ‘leave‐one‐out’ sensitivity analysis was employed to assess whether the results were influenced by any single SNP.

### 2.2 Selection of Instrumental Variables

Three MR assumptions need to be fulfilled for the selection of SNPs as IV: (1) the instrumental variable should be correlated with exposure (i.e., 1400 metabolite traits); (2) the instrumental variable should be independent of outcome (HV‐related traits); and (3) the instrumental variable cannot affect the outcome in any way other than exposure (i.e., there is no horizontal polytomous validity). Significant gene loci independently associated with SMs (*p* < 1 × 10^−5^) were first screened. To remove Link‐age disequilibrium (LD), the r threshold for the linkage disequilibrium parameter of IV was set to 0.001 and the genetic distance window was set to 10,000 kb (Auton et al. 2015). F‐statistics were calculated for each SNP, data with F‐statistics < 10 were excluded, and the remains could be used as a strong variable IV to exclude outliers by MR pleiotropy residual sumand outlier (MR‐PRESSO) test (Yu et al. 2022a; Verbanck et al. 2018).

To rule out weak instrument bias for the metabolites screened as being associated with hallux valgus, this study used Chronotype (morningness/eveningness preference, GWAS ID: ieu‐b‐4862) as the negative control outcome for MR analysis. The feasibility of selecting chronotype was based on the following considerations: (1) Chronotype is primarily regulated by circadian rhythm genes (CLOCK, PER, CRY, etc.) and has no known direct cross‐regulation mechanisms with metabolic pathways, indicating biological independence; (2) The heritability of chronotype is estimated to be 21%–54%, and its genetic determination is mainly concentrated in circadian rhythm‐related loci, with minimal overlap with metabolic loci; (3) Chronotype has no known association with hallux valgus; (4) The GWAS includes 205,527 European ancestry participants, providing sufficient sample size and high SNP coverage. MR analyses were conducted using the IVW method, weighted median method, Simple mode, Weighted mode, and MR‐Egger method, followed by heterogeneity testing (Cochran's Q test) and horizontal pleiotropy testing (MR‐Egger intercept test).

### 2.3 Identification of Intersecting Genes and GO/KEGG Analysis

SMs with statistical significance (*p* < 0.05) by Weighted median, IVW, and Weighted mode were used to predict their targets of action through DrugBank (https://go.drugbank.com/), SuperPred (https://prediction.charite.de/), Genecards (https://www.genecards.org/), and SwissTargetPrediction (http://swisstargetprediction.ch/) databases to predict their targets of action; based on the Genecards database to predict HV‐related targets, and the SMs‐HV intersection targets were obtained using the Microbiotics online platform (http://www.bioinformatics.com.cn/). The obtained intersection targets were imported into the DAVID online platform (https://david.ncifcrf.gov/) for GO and KEGG analysis to further understand and elaborate the potential mechanism between positive SMs and HV.

### 2.4 Candidate Gene Protein–Protein Interaction Network Construction

SMs‐disease intersection targets were imported into the STRING (https://cn.string‐db.org/) platform and, after exporting as tsv files, PPI networks were constructed using Cytoscape 3.9.1 software.

### 2.5 MR Analysis to Screen for Key Genes

The obtained SMs‐disease intersection targets were used as exposure factors, and SMs and HV, which were statistically significant by all three methods, were analyzed separately by MR as endpoints. The key targets with causal relationships with both SMs and HV were selected for subsequent prediction and identification of relevant compounds.

### 2.6 Experimental Validation of Serum and Tissue From HV Patients

#### 2.6.1 Collection of Clinical Samples

Thirty‐six HV patients admitted to the Foot and Ankle Surgery Department of Affiliated Orthopedic Hospital of Chongqing University of Traditional Chinese Medicine from June 2023 to January 2024 who met the indications for surgery were included in the HV group, and another 36 patients who required surgical treatment due to trauma were included in the Control group. In the HV group, there were 6 males and 30 females, aged 23–75 years, 21 bipedal HVs and 15 unipedal HVs. In the Control group, there were 10 males and 26 females, aged 25–74 years. The mean age, mean height, mean weight, and mean body mass index of the two groups of patients were compared, and the results were not statistically significant (*p* > 0.05), as shown in Table 1.

**TABLE 1:** Comparison of general information of patients in two groups.

Groups	*n*	Average age (Years)	Average weight (Kg)	Average height (Cm)	Average body mass index
HV groups	36	56.97 ± 2.62	59.47 ± 7.53	164.51 ± 3.13	22.37 ± 2.22
Control groups	36	57.47 ± 2.16	60.89 ± 7.66	163.72 ± 3.50	23.17 ± 2.54
*t*	—	0.883	0.793	1.009	1.422
*p*	—	0.380	0.430	0.316	0.159

To ensure that SMs levels reflect the patients' baseline status rather than an acute stress response, all blood samples were collected during a stable phase of the patient's condition. For the HV group, samples were obtained after admission but before elective surgery, at a time when the patients showed no acute infection or inflammatory exacerbation. For the Control group, samples were also collected prior to elective surgery once the patients had passed the acute phase of trauma (typically 48–72 h post‐injury), were haemodynamically stable, and exhibited no fever or signs of systemic inflammatory response syndrome. Prior to blood collection, none of the patients had received blood transfusions, parenteral nutrition, or any medication that could interfere with metabolism, thereby ensuring that the baseline data of the Control group were representative.

#### 2.6.2 Enzyme‐Linked Immunosorbent Assay (Elisa)

Whole blood samples were drawn from patients who met the experimental criteria, left to stand and then centrifuged at 3000 rpm for 10 min at 4°C. The serum was extracted and stored at −80°C for subsequent experiments. The levels of differential metabolites in the sera of the two groups of patients were further verified by measuring the absorbance at 450 nm using the Infinite 200 PRO ELISA (Life Sciences, USA) using the Human Cysteine ELISA kit (MM‐60026H1) (Meiman, China).

#### 2.6.3 Quantitative Reverse Transcription‐Polymerase Chain Reaction (qRT‐PCR)

The expression levels of key targets in bone tissue were further verified in both groups of patients by qRT‐PCR (Applied Biosystems, USA). Relative gene expression levels were normalized to Gapdh according to the $2^{-\Delta \Delta C_{T}}$ method. Primer sequences used are shown in Table 2.

**TABLE 2:** The primers used in this study.

Gene	Forward primer	Reverse primer
FGFR2	AGCACCATACTGGACCAACAC	GGCAGCGAAACTTGACAGTG
Gapdh	TGTGGGCATCAATGGATTTGG	ACACCATGTATTCCGGGTCAAT

### 2.7 Prediction of Active Ingredients and Validation of Molecular Docking and Molecular Dynamics Simulation of Natural Marine Drugs

Screening was performed in the Comprehensive Marine Natural Products Database (CMNPD) (https://www.cmnpd.org) to identify potential active compounds associated with key targets. SDF files of small molecule ligands were downloaded in the PubChem (https://pubchem.ncbi.nlm.nih.gov/) database; PDB files of protein crystal structures of key genes were downloaded in the RCSB Protein Database (https://www.rcsb.org/). AutoDock Vina, AutoDock Vina, Pymol, and Openbabel 3.1.1 were used for molecular docking and visualization. Pymol was used to remove water molecules and the rest of the ligands from the large molecule receptors, and Openbabel 3.1.1 was used for the conversion of small molecules into SDF format. All were saved in PDB format and then nine molecular dockings were performed using AutoDock Vina. The ligand‐receptor complex files in PDBQT format were imported into Pymol for optimisation and adjustment to obtain molecular docked conformations. MD simulations were performed based on GROMACS 2020.1 software. CHARMM 36 field was used for the protein system, TIP3P was used for the water molecule model, and the simulation box was a dodecahedral‐water box with the complexes at least 1.0 nm away from the edge of the box. NVT equilibrium: 100 ps; NPT equilibrium: 100 ps. In the present study, various parameters were calculated to analyze the protein‐compound binding mode and the change in the tightness of the protein in the presence or absence of the ligand in terms of Root Mean Square Deviation (RMSD), Root Mean Square Fluctuation (RMSF), Radius of Gyration (Rg) curves and Hydrogen bonding.

### 2.8 Statistical Methods

In this study, the analysis was implemented using R (v 4.2.1) and R packages such as TwoSampleMR, MendelianRandomisation, MRPRESSO, etc. The results of the analyses were corrected using the False Discovery Rate (FDR) method, which defines *p* ≤ 0.05 and *q*‐value ≤ 0.2 as the threshold for significant causality, and *p* ≤ 0.05 but *q*‐value > 0.2 was considered suggestive of association (Liu et al. 2024). Statistical analyses and histograms from the results were plotted using GraphPa Prism 8 software. All data are expressed as mean ± standard deviation. An unpaired *t*‐test was used for comparison between the two groups.

## 3 Results

### 3.1 Causal Relationship Between Two SMs and HV

**FIGURE 1:**
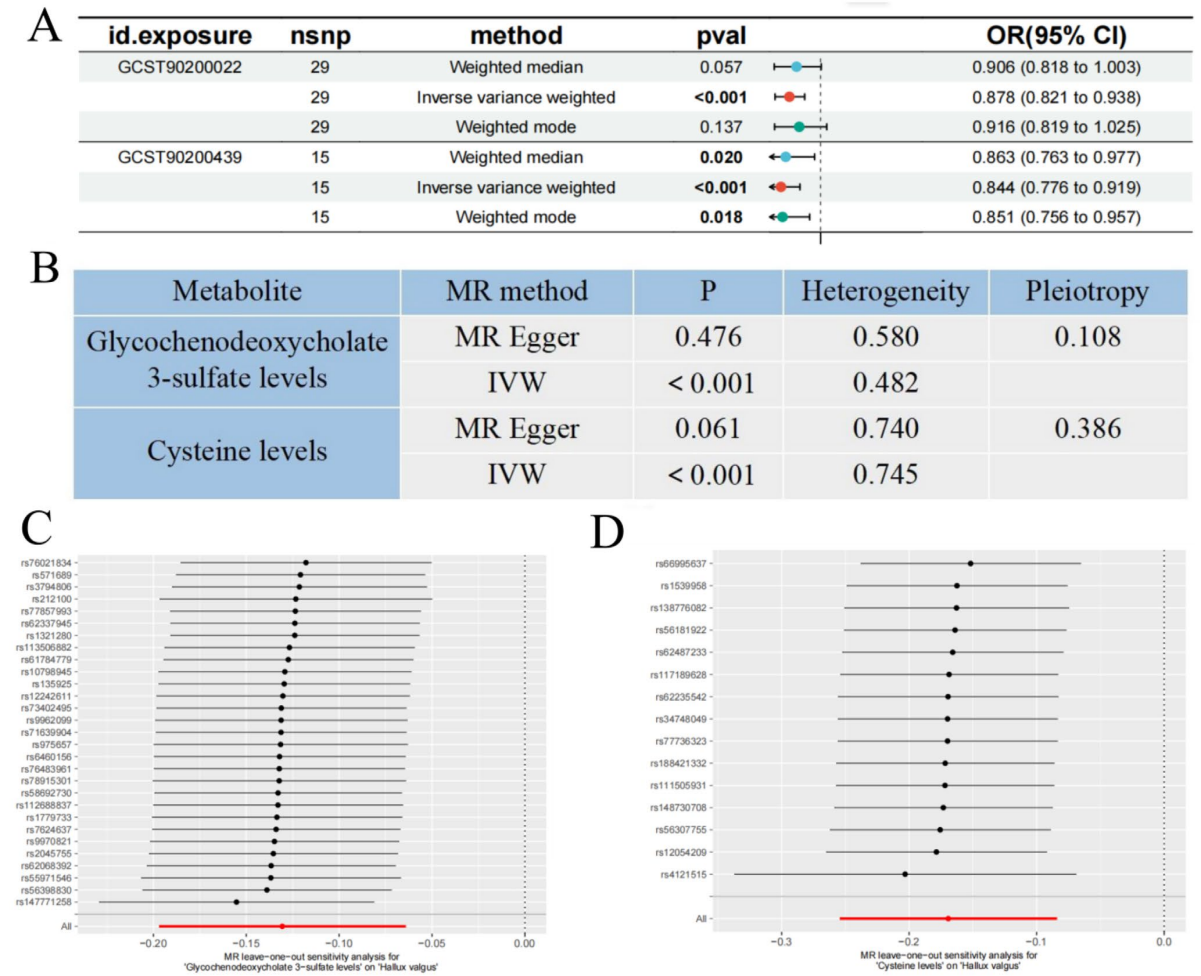
Causal relationship and test for heterogeneity and pleiotropy between two metabolites and HV. (A) Forest plot of causality between Glycochenodeoxycholate 3‐sulfate, Cysteine and HV; (B) Results of heterogeneity and multiplicity test between Glycochenodeoxycholate 3‐sulfate, Cysteine and HV; (C, D) Sensitivity analysis plots of Glycochenodeoxycholate 3‐sulfate, Cysteine and HV.

A total of 1400 SMs were analyzed in this study, of which 2 metabolites were identified to be significantly correlated with HV based on the IVW model (Figure 1A), namely Glycochenodeoxycholate 3‐sulfate [OR (IVW) = 0.878, OR (95% CI) 0.821–0.938, *p* < 0.001], Cysteine [OR (IVW) = 0.844, OR (95% CI) 0.776–0.919, *p* < 0.001]. The levels of both serum metabolites negatively affected HV. As shown in Figure 1B, neither heterogeneity nor pleiotropy was detected for Glycochenodeoxycholate 3‐sulfate or Cysteine. In addition, leave‐one‐out sensitivity analyses were performed for both metabolites (Figure 1C,D). No single SNP was found to disproportionately influence the results, indicating that the findings were robust.

### 3.2 No Causal Relationship Between HV and the Two SMs

**FIGURE 2:**
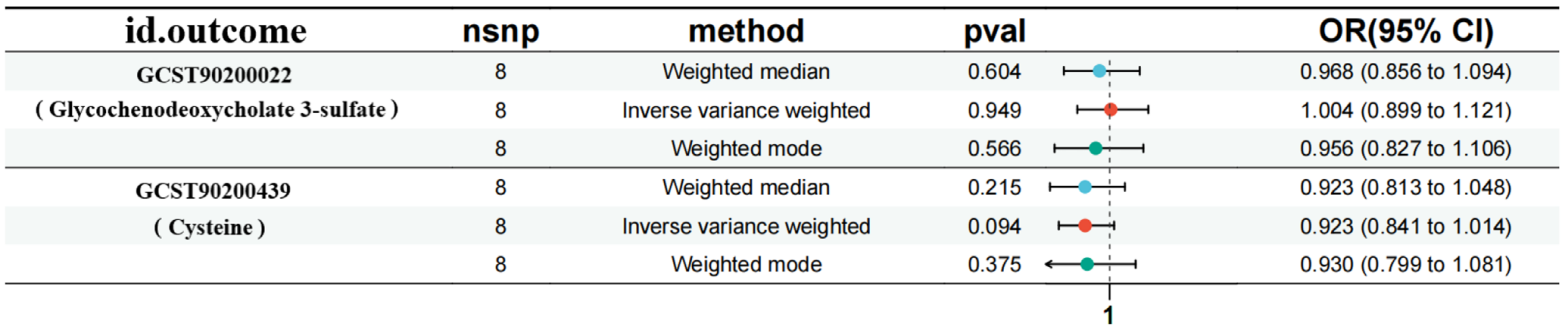
Forest plot of reverse causality between HV and Cysteine, Glycochenodeoxycholate 3‐sulfate.

Using HV as the exposure factor, two metabolites, Cysteine and Glycochenodeoxycholate 3‐sulfate, were used as the endpoints for the inverse MR analyses (Figure 2). The results showed that Glycochenodeoxycholate 3‐sulfate [OR (IVW) = 1.004, OR (95% CI) 0.889–1.121, *p* = 0.949], Cysteine [OR (IVW) = 0.923, OR (95% CI) 0.841–1.014, *p* = 0.094], indicating that HV does not positively or negatively affect the levels of either SMs.

### 3.3 Negative Control Analysis Results

According to Figure 1, SMs (Cysteine) with statistical significance (*p* < 0.05) in Weighted median, IVW, and Weighted mode were selected for subsequent analysis. The negative control analysis using Chronotype as the outcome showed no significant causal association between Cysteine and Chronotype (Table S2, Figures S1–S4). The IVW method yielded an OR of 0.998 (95% CI 0.961–1.039, *p* = 0.978), consistent with the weighted median (*p* = 0.972), MR‐Egger (*p* = 0.511), Weighted mode (*p* = 0.922), Simple mode (*p* = 0.922) methods. Heterogeneity testing (Cochran's Q test, *p* = 0.084) and horizontal pleiotropy testing (MR‐Egger intercept test, *p* = 0.450) revealed no significant abnormalities. These null findings indicate that our instrumental variables are not substantially affected by weak instrument bias, supporting the robustness of the primary finding.

### 3.4 Identification of Intersecting Targets and GO/KEGG Analysis

**FIGURE 3:**
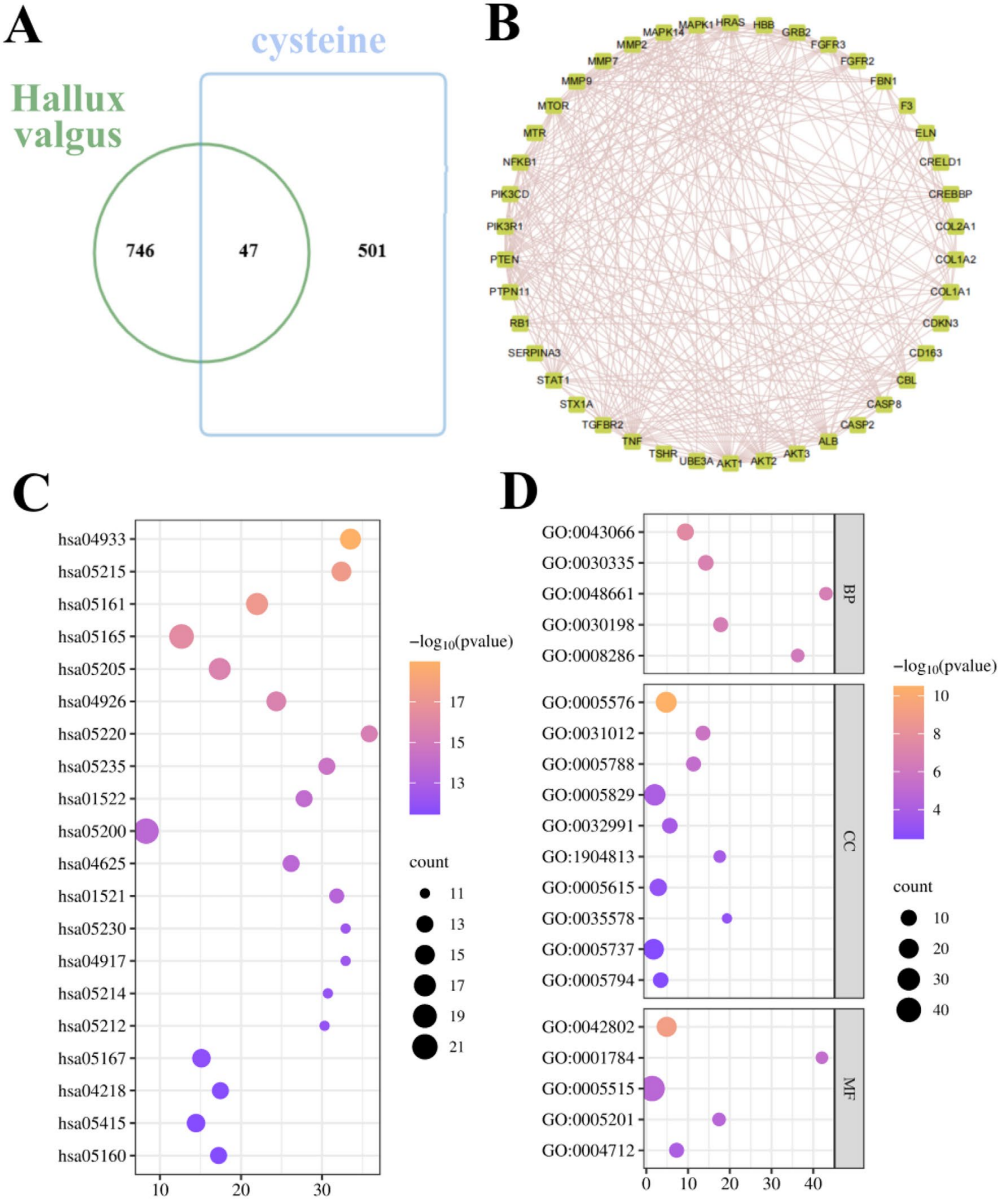
Intersection target identification and enrichment analysis. (A) VENN plot of 793 HV‐associated targets versus 548 Cysteine‐associated targets; (B) PPI network plot of 47 intersecting targets; (C) KEGG‐enriched bubble plot of 47 intersecting targets; (D) GO‐enriched bubble plot of 47 intersecting targets.

A total of 548 Cysteine‐related targets were obtained from four databases, including DrugBank and SuperPred, and 793 HV‐related targets were obtained from the Genecards database, which contained 47 intersecting targets (Figure 3A). 42 targets and 287 edges were contained in the PPI network graph (Figure 3B). KEGG enrichment results showed that the 47 intersections 793 involved in hsa04218 (Cellular senescence), hsa04933 (AGE‐RAGE signaling pathway in diabetic complications) and other pathways (Figure 3C). The results of the GO enrichment analysis showed that the intersected genes were mainly involved in GO:0043066 (negative regulation of apoptotic process) and other biological processes and GO:0005576 (extracellular region) and other cellular components (Figure 3D).

### 3.5 Fibroblast Growth Factor Receptor (FGFR) 2 Demonstrates a Causal Relationship With Both Cysteine and HV

**FIGURE 4:**
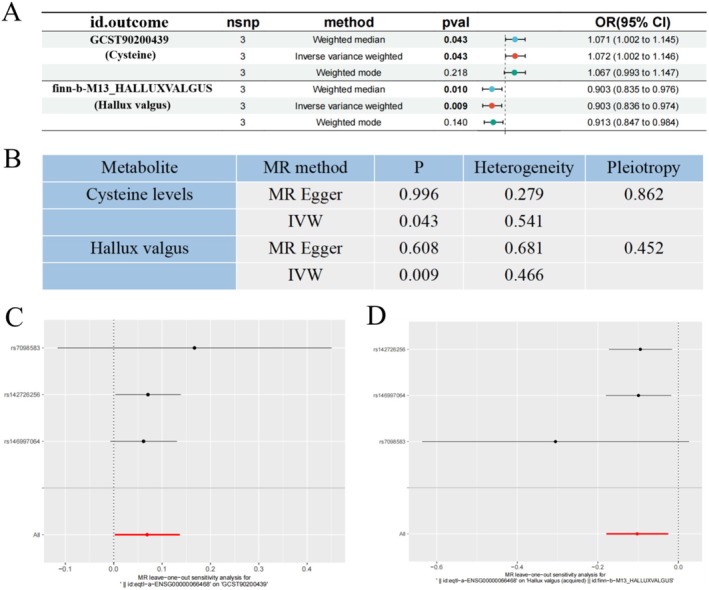
Causality and test of heterogeneity and pleiotropy between FGFR2 and Cysteine and HV. (A) Forest plot of causality between FGFR2 and Cysteine and HV; (B) results of heterogeneity and multiplicity tests between FGFR2 and Cysteine and HV; (C, D) sensitivity analyses plots of FGFR2 versus Cysteine and FGFR2 versus HV.

47 intersecting targets as exposure factors were analyzed by SMR with Cysteine and HV, respectively, and FGFR2 had a positive causal relationship with Cysteine and a negative causal relationship with HV. See Figure 4A, a significant correlation between FGFR2 and Cysteine and HV was determined based on the IVW model for Cysteine [OR (IVW) = 1.071, OR (95% CI) 1.002–1.145, *p* = 0.043], HV [OR (IVW) = 0.903, OR (95% CI) 0.836–0.974, *p* = 0.009]. Therefore, FGFR2 was regarded as a key target for subsequent studies. See Figure 4B, neither Heterogeneity nor Pleiotropy was observed between FGFR2‐Cysteine and FGFR2‐HV. sensitivity analyses were also performed using ‘leave‐one‐out’, and neither FGFR2‐Cysteine nor FGFR2‐HV did not show any SNPs that significantly affected the results, and the results were robust (Figure 4C,D).

### 3.6 Validation of the Key Metabolite Cysteine Against the Key Target FGFR2

**FIGURE 5:**
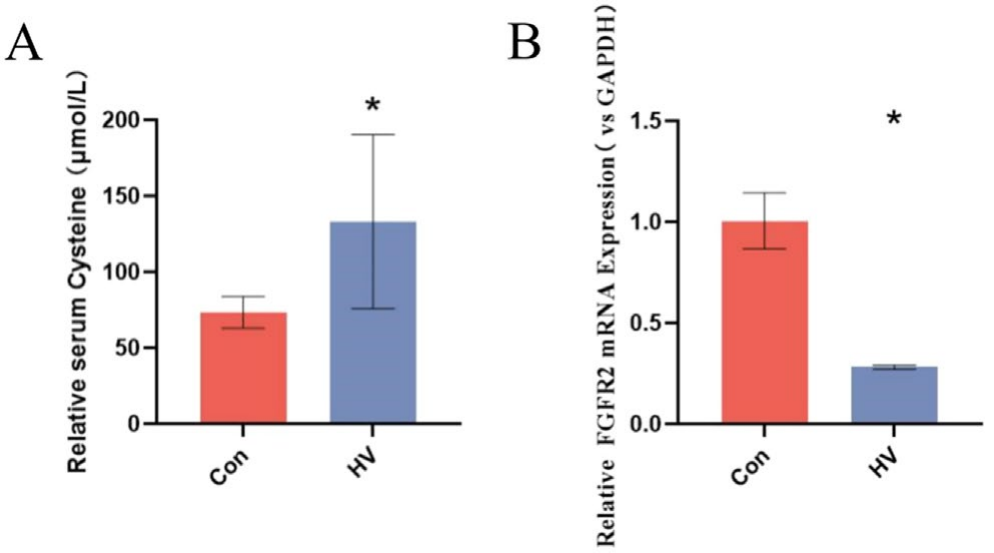
Validation of Elisa and qRT‐PCR quantitative analysis. (A) Serum Cysteine index level of patients in both groups; (B) FGFR2 mRNA expression in bone tissue of patients in both groups; *p < 0.001 compared with Con group.

The metabolic level of Cysteine in serum of the two groups was detected by Elisa method, and the results showed that the serum Cysteine level of the patients in the HV group was significantly lower compared with that of Con (*p* < 0.001) (Figure 5A); and the expression level of the FGFR2 gene in the bone tissue of the patients in the two groups was detected by qRT‐PCR method, and the results showed that the patients in the HV group had a significantly lower (*p* < 0.001) expression level of the FGFR2 gene in the bone tissue compared with Con (Figure 5B).

### 3.7 Prediction of Active Ingredients and Validation by Molecular Docking and Kinetic Simulation

**FIGURE 6:**
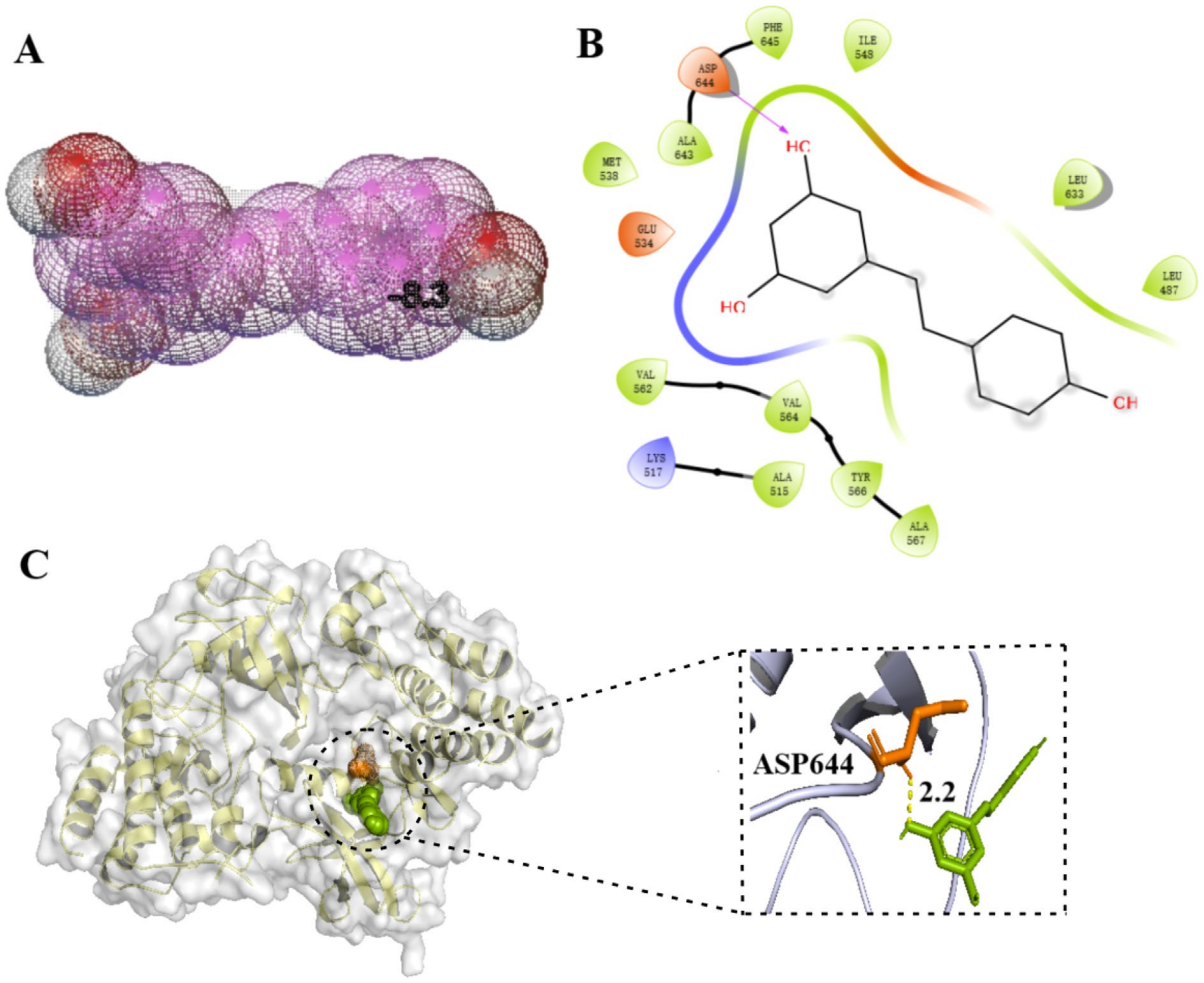
FGFR2‐Isogranulatimide docking best conformation and 2D and 3D visualization. (A) Best conformation of FGFR2‐Isogranulatimide docking with a binding energy of −8.3 Kcal/mol; (B) 2D docking diagram of the best conformation; (C) 3D docking diagram of the best conformation.

The CMNPD database was searched for FGFR2‐associated natural marine pharmaceutical active ingredients, i.e., Isogranulatimide, which was molecularly docked as a small‐molecule ligand and FGFR2 as a receptor macromolecule. Figure 6A shows the best docking conformation of FGFR2‐Isogranulatimide with a binding energy of −8.3 Kcal/mol. Figure 6B,C how the binding of FGFR2‐Isogranulatimide from 2D and 3D angles, respectively. Based on the results, it can be seen that the FGFR2 amino acid residue ASP‐644 can bind to Isogranulatimide through hydrogen bonding interaction force at a distance of 2.2 Å.

**FIGURE 7:**
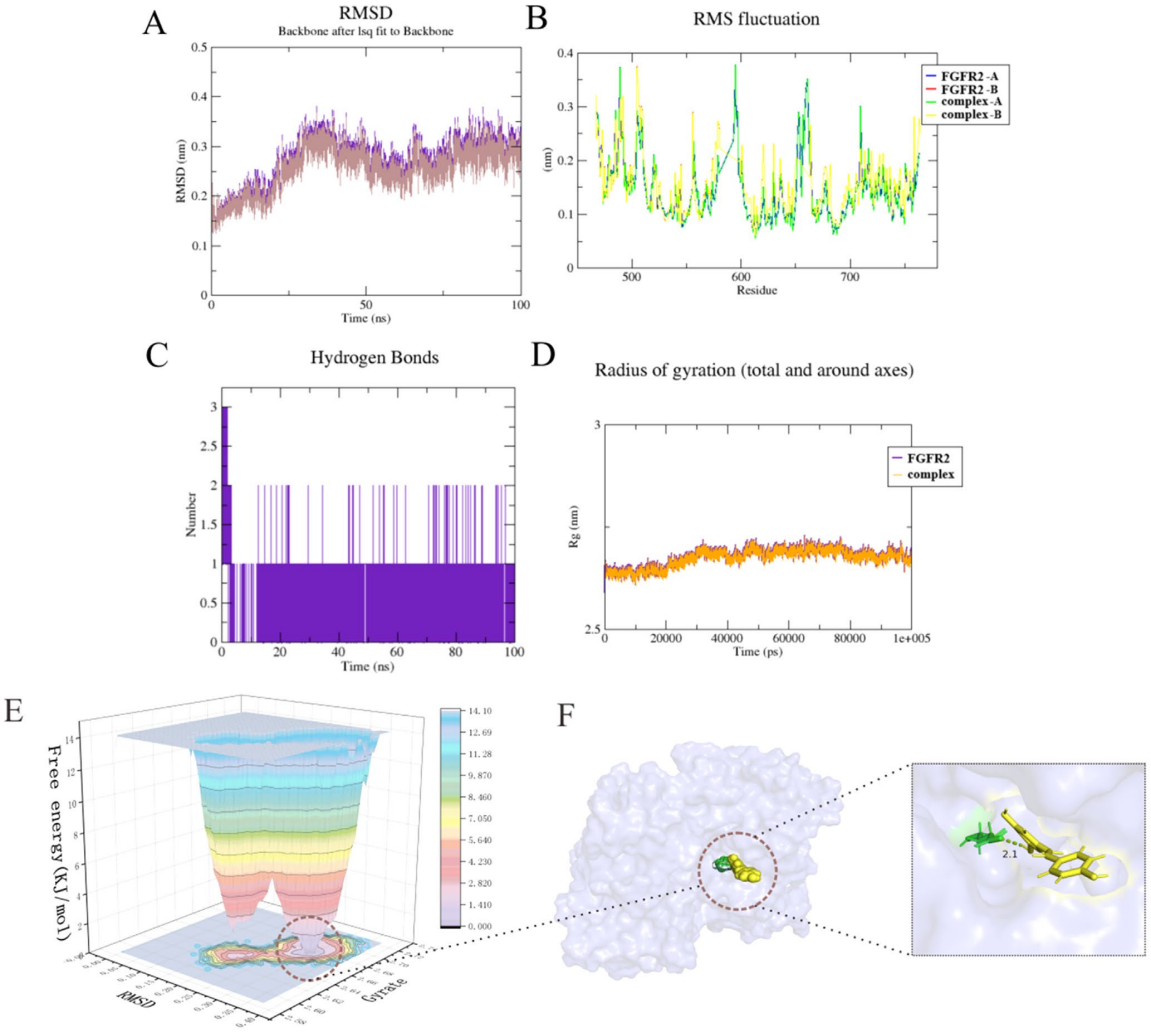
Molecular dynamics simulations of FGFR2‐Isogranulatimide. (A) free FGFR2 backbone‐complex RMSD plot; (B) FGFR2‐complex RMSF plot; (C) complex hydrogen bonding plot; (D) FGFR2‐complex Rg plot; (E) complex FEL plot; (F) optimal conformation in the energy‐poor region.

The above molecular docking results indicate that the FGFR2‐Isogranulatimide complex has a strong binding affinity. The stability and conformational changes of the complex were further analyzed by 100 ns MD simulations. It is generally accepted that a smaller RMSD value represents a more stable conformation. As shown in Figure 7A, the free FGFR2 backbone and Isogranulatimide complex had no obvious separation and breakage during the whole simulation process, and their RMSD values had no obvious difference in the pre‐simulation period, and gradually reached a stable state after 40 ns. This result indicates that the FGFR2 backbone does not lead to substantial changes in itself when binding occurs with the ligand, and the ligand can be firmly attached to the FGFR2 backbone. The RMSF plot shows the changes in amino acid residues of the protein. As shown in Figure 7B, FGFR2 consists of two chains, Chain A and Chain B. Most of the simulations caused minor fluctuations in the amino acid structure, and very few of them showed slight structural changes. The RMSF values of the two chains of FGFR2 and the complexes, Chain A and Chain B, were ≤ 0.4 nm and most of them showed stable fluctuations around 0.1 nm. The results suggest that the binding of FGFR2‐Isogranulatimide may cause a small range of protein residue fluctuations, but the overall stability is high. The number and density of hydrogen bonds in the complexes can reflect their binding strength to some extent. As shown in Figure 7C, the number and density of hydrogen bonds and the strength of FGFR2‐Isogranulatimide complexes are high. The maximum number of hydrogen bonds formed between the complexes was up to 3 at around 1 ns, with high binding stability. The results of Rg analysis showed that the rotational radii of both free FGFR2 and the complexes were relatively stable, indicating that the protein conformation would not be affected by Isogranulatimide (Figure 7D). The free energy landscape (FEL) characterizes the changes in free energy experienced by a substance during the simulation process. By calculating the free energy landscape of protein and ligand complexes it is possible to give guidance on the characteristic conformation of the extracted complexes. The darker the color in the FEL, the lower the binding energy. The FEL for the FGFR2‐Isogranulatimide complex was established by combining the data of both RMSD and Rg. As shown in Figure 7E, during the simulation, there is mainly one energy low‐lying region on the free energy map, indicating a relatively stable state in the complex structure. The conformations in the energy low‐lying region were extracted for visualization (Figure 7F).

## 4 Discussion

In this study, we found a significant negative causal relationship between Cysteine and HV in 1400 SMs, i.e., the risk of developing HV may increase when Cysteine levels are decreased. To test the reliability of this result, we examined the variability of Cysteine levels by collecting serum from patients, and the results showed that serum Cysteine levels were significantly lower in HV patients than in non‐HV patients. This result provides some support for the SMR analysis described above. In addition, we used various databases to predict the relevant targets of Cysteine and HV, where 47 Cysteine‐HV intersection targets were obtained after taking the intersection, carrying out GO and KEGG enrichment analysis and constructing the PPI network diagram. The causal relationship between the 47 intersected targets and Cysteine and HV was analyzed, and it was clarified that there was a positive causal relationship between FGFR2 and Cysteine, while there was a negative causal relationship with HV. Bone tissues of patients were also collected for validation, and the results showed that FGFR2 levels in bone tissues of HV patients were significantly lower than those of non‐HV patients. Finally, we predicted Isogranulatimide, a natural marine drug active ingredient with a therapeutic effect on HV, based on FGFR2. The results of molecular docking and molecular dynamics simulation showed that FGFR2‐Isogranulatimide had good binding energy, suggesting that Isogranulatimide might be able to affect HV by regulating FGFR2 levels.

Fibroblast growth factors (FGFs) and their receptors (FGFRs) control a wide range of biological functions, regulating cell proliferation, survival, migration, and differentiation, and play an important role in organ formation as well as bone growth (McIntosh et al. 2000; Su et al. 2014; Turner and Grose 2010). Mutations in FGFRs cause various disorders of human skeletal dysplasia, such as chondrodysplasia syndrome and premature closure of the cranial sutures syndrome (Chen and Deng 2005; Rousseau et al. 1994). FGFR2 is a receptor tyrosine kinase belonging to the immunoglobulin superfamily, which is mainly involved in cell proliferation, differentiation, survival, and migration, and regulates signaling pathways including RAS‐MAPK (ERK1/2), PI3K‐AKT, PLCγ‐PKC, and signal transducers and activators of transcription (Ornitz and Itoh 2015). FGFR2 plays a critical role in many biological processes, including embryonic development, tissue repair, and tumor formation. Previous studies have found that FGF2 is widely expressed in epithelial, osteoblast, and chondrocyte lineages (Yang et al. 2019; Britto et al. 2001), is an important regulator of osteoclast–osteoblast interactions or coupling, and plays an important role in the maintenance of bone homeostasis, particularly in endochondral bone formation (Montero et al. 2000; Lazarus et al. 2007; Zhang et al. 2021; Ornitz and Marie 2015). This process of receptor‐ligand binding and signaling is an integral part of biological processes such as skeletal development, limb and skin formation, and the formation of various organs (Lomri et al. 1998; Xu et al. 1999; Zhu et al. 2023).

HV is hereditary and can be manifested especially in different populations and genders (Hsu et al. 2015; Hannan et al. 2013). According to previous studies, 63%–90% of patients with HV have a family history of HV, but there are few studies on the genetics of HV. Genetic factors that lead to metatarsal abnormalities and thus HV most commonly include four types: rounded first metatarsal head, excessive intermetatarsal angle, metatarsal inversion, and congenital anomalies of the seed bones (Nix et al. 2012; D'Arcangelo et al. 2010). In the study of Jia et al., it was found that genes such as Titin, Collagen type VI alpha 3 chain, and Leucyl‐tRNA synthetase are closely related to HV, and these genes have been shown to play an important role in the anatomical development of long toes and long fingers (Jia et al. 2021). Whereas FGFR2 is expressed in the condensed mesenchyme of early limb buds, human long bones are formed by endochondral ossification (Chen and Deng 2005; Su et al. 2008). The molecular basis of these pathological changes may be attributed to disrupted signaling pathways resulting from reduced Cysteine availability. Cysteine is essential for maintaining the structural integrity of FGFR2 through the formation of disulfide bonds in its extracellular domain (Robertson et al. 1998; Mangasarian et al. 1997). When Cysteine levels are insufficient, impaired disulfide bonding may compromise FGFR2 dimerisation and activation upon ligand binding. This disruption predominantly affects two key downstream cascades: the RAS‐MAPK (ERK1/2) and PI3K‐AKT pathways (Li et al. 2024, 2025), both of which are critical for chondrocyte survival, osteoblast differentiation, and extracellular matrix synthesis.

Specifically within the first metatarsophalangeal joint, attenuated MAPK signaling may reduce collagen type II and aggrecan production in articular cartilage (Li et al. 2024), accelerating degeneration under mechanical stress. Concurrently, diminished PI3K‐AKT activity in osteoblasts could impair subchondral bone formation, weakening the structural support of the metatarsal head. These changes contribute to the destabilization of the first ray and the progressive valgus deformity. Regarding ligamentous structures, reduced FGFR2 signaling in fibroblasts of the medial collateral ligament may decrease the expression of collagen type I and elastin (Hankemeier et al. 2005), compromising tensile strength and leading to progressive ligamentous laxity. This laxity permits further lateral deviation of the proximal phalanx, perpetuating the deformity. Furthermore, insufficient FGFR2 activity in the medial eminence of the first metatarsal head might compromise the formation of protective osteochondral structures (Wernlé et al. 2023), thereby facilitating the development of the characteristic bony prominence (Lories and Luyten 2007). Thus, the Cysteine‐FGFR2 axis integrates metabolic status with joint tissue integrity, offering a mechanistic link between low serum Cysteine and the characteristic soft tissue and bone changes observed in HV.

It is important to contextualize these mechanistic insights within the current clinical management of HV. Conservative treatment, including footwear modification and foot orthoses, remains the first‐line approach for most patients, particularly those with mild‐to‐moderate deformity (Colò et al. 2024; Hurn et al. 2022). A recent comprehensive review by Colò et al. highlights that proper footwear, characterized by adequate length, a wide toe box, cushioned sole, and lowered heel, can alleviate symptoms and improve patient comfort, although there is no consensus that such interventions can correct the deformity or halt its progression (Colò et al. 2024). Foot orthoses, particularly those addressing subtalar joint hyperpronation, may play a role in controlling HV progression, yet evidence regarding their efficacy in achieving structural correction remains variable (Colò et al. 2024; Hurn et al. 2022).

These observations underscore the need for adjunctive or alternative strategies that target the underlying biological processes driving HV pathogenesis. Our findings suggest that modulating Cysteine levels or targeting FGFR2 signaling could represent a novel approach to complement existing conservative measures. For instance, in early‐stage HV where biomechanical stressors initiate joint instability, interventions aimed at maintaining adequate Cysteine levels might preserve FGFR2 structural integrity and function, thereby supporting cartilage homeostasis and ligamentous strength. Such metabolic modulation could potentially delay disease progression or enhance the efficacy of orthotic devices by maintaining the biological resilience of joint tissues. Future research should explore whether combining conservative biomechanical interventions with metabolic or targeted pharmacological approaches offers synergistic benefits, particularly for patients at risk of progression despite optimal non‐surgical management. However, translating Cysteine modulation into a clinical therapeutic strategy requires careful consideration of its bioavailability, safety, and potential risks. Direct supplementation of Cysteine is often limited by its rapid metabolism and auto‐oxidation in the gastrointestinal tract. Therefore, stable precursors like N‐acetylcysteine (NAC), which exhibit superior oral bioavailability and established safety profiles, are typically preferred in clinical settings (Pedre et al. 2021). While NAC is generally well‐tolerated, high‐dose or long‐term systemic administration may induce adverse effects, including gastrointestinal disturbances, and potentially alter the systemic redox balance. Furthermore, because Cysteine is fundamentally involved in numerous pleiotropic metabolic pathways across various tissues, systemic elevation could theoretically lead to off‐target effects (Tenório et al. 2021). Future investigations must not only establish optimal and safe dosing regimens but also explore joint‐targeted delivery systems (e.g., intra‐articular injections or localized hydrogels) to maximize local therapeutic efficacy at the first metatarsophalangeal joint while minimizing systemic exposure risks (Zhao et al. 2025).

In studies on HV, it has been found that secreted FGF interferes with driving the differentiation of mesenchymal cells present in the soft tissues covering the bunion into bone, which further forms a bone mass or stabilizes the bunion (Zhang et al. 2021; Ornitz and Marie 2015; Yu and Ornitz 2008; Verheyden et al. 2005). It is well known that bony encumbrances arise in the junction area where the synovium covers the bone. There is no evidence to suggest that bony encumbrances cause signs and symptoms in the surrounding joints. On the contrary, the appearance of bone recession reflects the body's efforts to repair damaged tissues and maintain joint stability in the face of damaged joints (Lories and Luyten 2007). In our study, we found that there is a negative causal relationship between FGFR2 and HV, and we speculate that in the case of FGFR2 deficiency, the bunion deformity is formed due to the inability to promote bone formation in time to prevent the onset or progression of the bunion deformity (Xiao et al. 2010). And after a long period of bunion deformity, and then new problems such as inflammatory irritation further promote bone formation.

Cysteine is an amino acid that plays an important role in organisms as the basic building block of proteins. It is involved in a variety of biochemical processes such as redox reactions and metal binding (Poole 2015). Cysteine has an important role in the structure and function of proteins because its chemical structure contains a sulfur atom (Woycechowsky and Raines 2003). One of its important functions is the formation of disulfide bonds, which promotes the correct folding and stability of proteins. The formation of this disulfide bond is crucial for protein function as it determines the three‐dimensional structure of the protein, which in turn affects its functional properties (Freedman 1989). In FGFR2, the disulfide bond formed by Cysteine helps to maintain the tertiary and quaternary structure of the receptor, thus ensuring its structural stability and correct folding, which is essential for the functioning of FGFR2 (Robertson et al. 1998; Neilson and Friesel 1995). Cysteine in the exocytotic region is involved in the process of receptor dimerisation upon ligand binding, which is important for FGFR2 activation and signaling (Sarabipour and Hristova 2016). The binding of ligands (e.g., FGF) induces two FGFR2 molecules to form a dimer, which is highly stable through the disulfide bonds formed by Cysteine (Eswarakumar et al. 2005; Mohammadi et al. 2005). Mutations in the FGFR2 gene are often associated with a variety of diseases, including abnormal bone development and cancer (Robertson et al. 1998; Chang et al. 2006). These mutations typically affect Cysteine residues, leading to abnormal disulfide bond formation and thus affecting FGFR2 function. Overall, Cysteine performs a variety of important functions in organisms, including protein structure and function, redox reactions, and cell signaling.

Isogranulatimide is an interesting marine compound mainly extracted from marine organisms such as ascidian (Hénon et al. 2007). This compound has attracted a lot of attention from researchers in the field of medicine, especially in anticancer research, due to its unique structure and potential biological activity (Sturgeon and Roberge 2007; Rundle et al. 2001; Lavrard et al. 2015; Deslandes et al. 2012). In our study, Isogranulatimide was identified as an active ingredient of marine drugs related to FGFR2, and the results of molecular docking and kinetic simulation confirmed the good binding activity between FGFR2 and Isogranulatimide. However, this study also has several limitations. First, the therapeutic intervention effect of Isogranulatimide on HV patients was not further verified in vivo; therefore, the mechanism of action of this marine compound associated with the key target FGFR2 on HV needs to be further explored in follow‐up research. Second, due to the limited sample size of the currently available serum metabolite GWAS datasets, a relatively lenient significance threshold (*p* < 1 × 10^−5^) was used for selecting instrumental variables. This was necessary to ensure sufficient SNPs were available for subsequent sensitivity and pleiotropy analyses. Although we rigorously filtered the SNPs using an F‐statistic > 10 to mitigate weak instrument bias, the potential subtle influence of this lenient threshold on causal inference cannot be entirely ruled out. Third, the sample size for our clinical validation was relatively small. Although our results demonstrated high statistical significance (*p* < 0.001), a limited sample size can inherently restrict statistical power and the generalizability of the findings. Future larger‐scale, multi‐center clinical studies are required to further validate the relationship between Cysteine, FGFR2, and HV pathogenesis. Fourth, the use of trauma patients as the control group for our clinical validation represents another limitation. While obtaining bone tissue from healthy individuals is ethically prohibited, utilizing trauma patients introduces the potential for confounding due to residual trauma‐induced systemic inflammation or local tissue repair processes. Although we strictly collected samples only after the acute stress phase had passed to minimize these effects, the subtle impact of trauma on metabolite levels and gene expression cannot be completely excluded. Importantly, the primary causal conclusions of this study are drawn from large‐scale Mendelian Randomization, which inherently mitigates this clinical sampling bias.

Several important considerations regarding the bioavailability of Isogranulatimide must be addressed when contemplating its repurposing from an anticancer agent to a potential therapeutic for HV. Isogranulatimide is a cell‐permeable alkaloid that acts as a potent, reversible, and ATP‐competitive inhibitor of Chk1 (IC50 = 100 nM) and GSK‐3β (IC50 = 500 nM), with established solubility in DMSO (50 mg/mL) (Roberge et al. 1998; Jiang et al. 2004). Its cell permeability suggests potential for systemic distribution, but whether it can achieve effective concentrations specifically within bone tissue remains to be determined.

The ability of small molecules to penetrate bone tissue is a critical factor for therapeutic efficacy in orthopedic conditions. Studies on drug penetration into bone have demonstrated that various compounds, including antibiotics and small molecules, can reach bone interstitial fluid spaces and achieve therapeutic concentrations (Yin et al. 2025). However, factors such as bone composition, vascularity, and drug physicochemical properties significantly influence bone penetration (Yin et al. 2025). For Isogranulatimide to exert its potential therapeutic effects on HV via FGFR2 modulation, it would need to reach adequate concentrations within the first metatarsophalangeal joint structures, including articular cartilage, subchondral bone, and periarticular soft tissues.

The transition from a compound primarily investigated as a cancer checkpoint inhibitor to a potential therapeutic for HV falls within the paradigm of drug repurposing (Saranraj and Kiran 2025; Czechowicz et al. 2025). Drug repurposing offers the advantage of leveraging existing safety and pharmacological data, potentially accelerating development timelines and reducing costs (Saranraj and Kiran 2025). However, several challenges must be addressed: (1) safety profiles established for short‐term oncology use may not directly translate to chronic administration for a non‐life‐threatening musculoskeletal condition; (2) the specificity of Isogranulatimide for FGFR2 relative to its primary targets (Chk1 and GSK‐3β) would need careful evaluation; and (3) potential off‐target effects in bone and joint tissues require thorough investigation (Czechowicz et al. 2025).

Future research directions should include: (a) pharmacokinetic studies to determine whether systemically administered Isogranulatimide achieves therapeutic concentrations in bone and joint tissues, potentially using methods established for bone penetration assessment (Yin et al. 2025); (b) development of bone‐targeted delivery strategies, such as conjugation with hydroxyapatite‐binding peptides or encapsulation in bone‐targeted nanoparticles, which have shown promise for enhancing drug accumulation in skeletal tissues (Jahed et al. 2019; Kasugai et al. 2000); and (c) comprehensive safety evaluation in preclinical models of HV to assess both local and systemic effects. These investigations are essential before Isogranulatimide can be considered a viable candidate for clinical development in HV management.

## 5 Conclusion

In conclusion, this study provides a new perspective on the causal relationship between bunion and metabolites and the identification and examination of key markers, i.e., the absence of Cysteine levels may lead to the development of HV, and FGFR2 can either directly increase the incidence of HV through negative regulation or increase the incidence of HV by positively regulating Cysteine levels, and the results have a certain reference value for the clinical targeted diagnosis and treatment of bunion patients, the results have certain reference significance and value.

## Author Contributions

**Xuefeng Luo:** data curation, formal analysis. **Yu Zhou:** conceptualization, writing – review and editing, writing – original draft, methodology, investigation, supervision, validation. **Songchuan Su:** conceptualization, methodology, funding acquisition, project administration. **Xin Li:** writing – original draft, data curation, formal analysis, methodology, software, investigation, validation. **Peng Lu:** resources, data curation, methodology, software, investigation. **Liqi Ng:** writing – review and editing, visualization. **Wei Huang:** writing – review and editing, visualization, validation. **Min Huang:** conceptualization, methodology, software.

## Funding

The authors would like to thank the Chongqing Yuzhong District Basic Research and Frontier Exploration Project (No. 20210142) for financial support.

## Ethics Statement

This study was approved by the Ethics Committee of Chongqing Orthopedic Hospital of Traditional Chinese Medicine (Approval No. GKYYIRB20240701, 11/7/2024). All of the experiments were performed in accordance with the Basel Declaration.

## Consent

The authors have nothing to report.

## Conflicts of Interest

The authors declare no conflicts of interest.

## Supporting information


**Figure S1:** Scatter plot of the Mendelian randomization analysis for the causal association between Cysteine and Chronotype.
**Figure S2:** Forest plot of the Mendelian randomization analysis for the causal effect of Cysteine on Chronotype.
**Figure S3:** Funnel plot of the Mendelian randomization analysis.
**Figure S4:** Leave‐one‐out sensitivity analysis plot for the Mendelian randomization analysis.

**Table S1:** STROBE‐MR checklist.

**Table S2:** Negative control analysis results of the causal association between Cysteine and Chronotype using Mendelian randomization.

## Data Availability

The datasets used and/or analyzed during the current study are available from the corresponding author on reasonable request.
