# Supplementary material for: Combining Mendelian Randomization and Experimental Validation to Reveal the Causal Relationship Between Hallux Valgus and Serum Metabolites and to Identify Their Therapeutic Targets and Relevant Components
Source: Food Sci Nutr. 2026 Jul 23;14(7):e72143. doi: 10.1002/fsn3.72143 (PMC13396431; doi:10.1002/fsn3.72143)
Supplement: Supplementary file 1 — Figure S1: Scatter plot of the Mendelian randomization analysis for the causal association between Cysteine and Chronotype. Figure S2: Forest plot of the Mendelian randomization analysis for the causal effect of Cysteine on Chronotype. Figure S3: Funnel plot of the Mendelian randomization analysis. Figure S4: Leave‐one‐out sensitivity analysis plot for the Mendelian randomization analysis. [file FSN3-14-e72143-s003.docx]

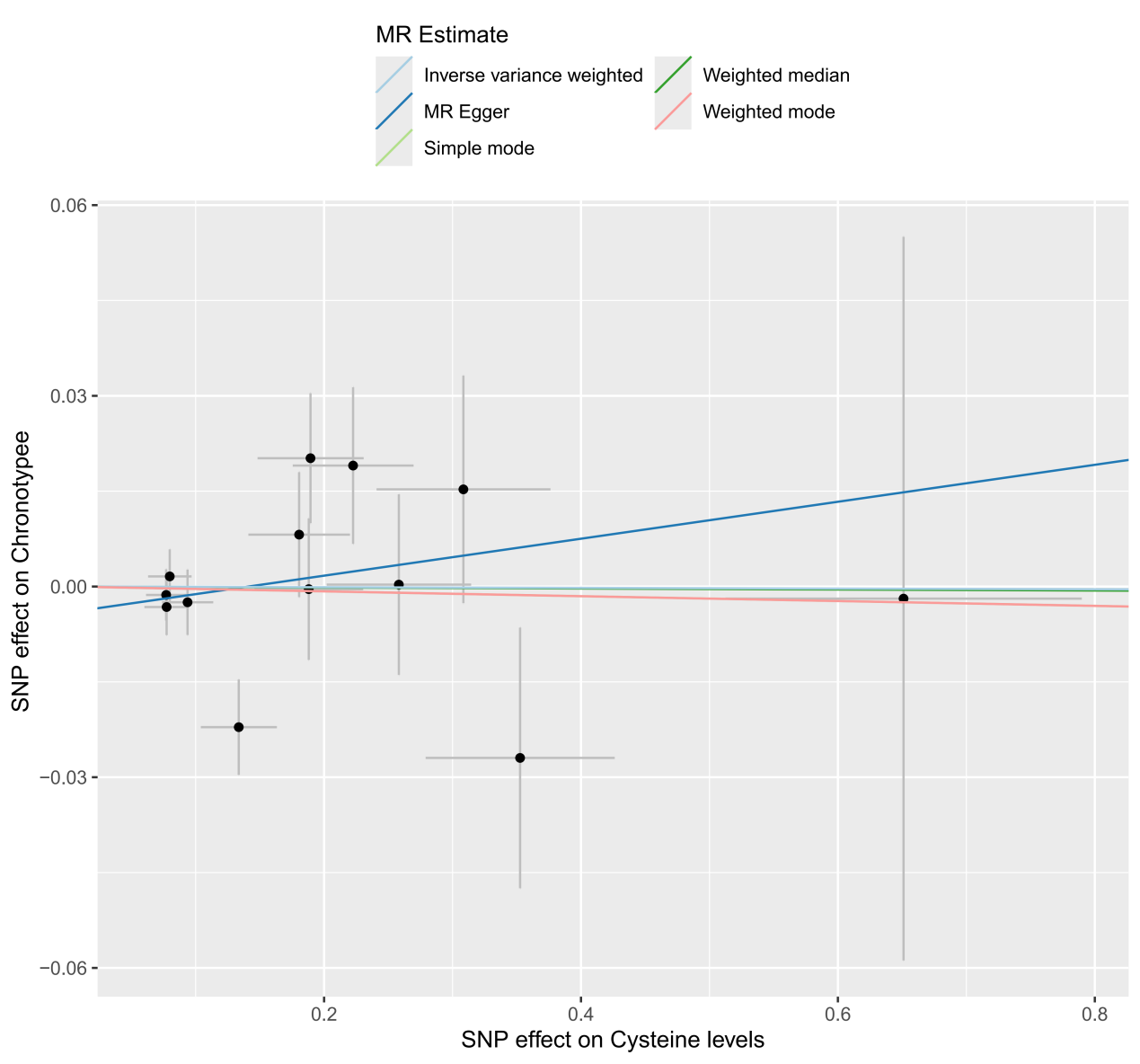


Supplementary Figure S1. Scatter plot of the Mendelian randomization analysis for the causal association between Cysteine and Chronotype.


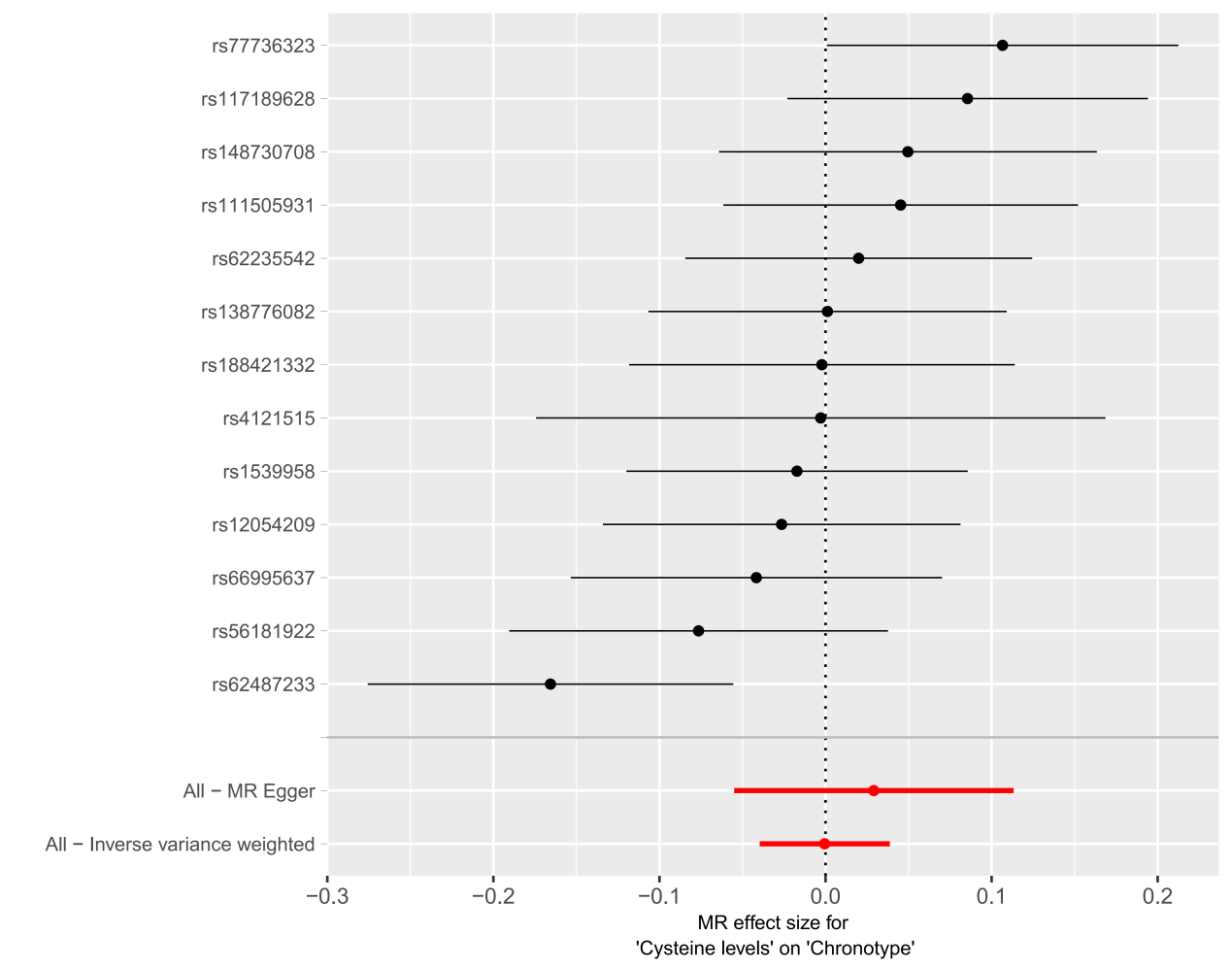


Supplementary Figure S2. Forest plot of the Mendelian randomization analysis for the causal effect of Cysteine on Chronotype.


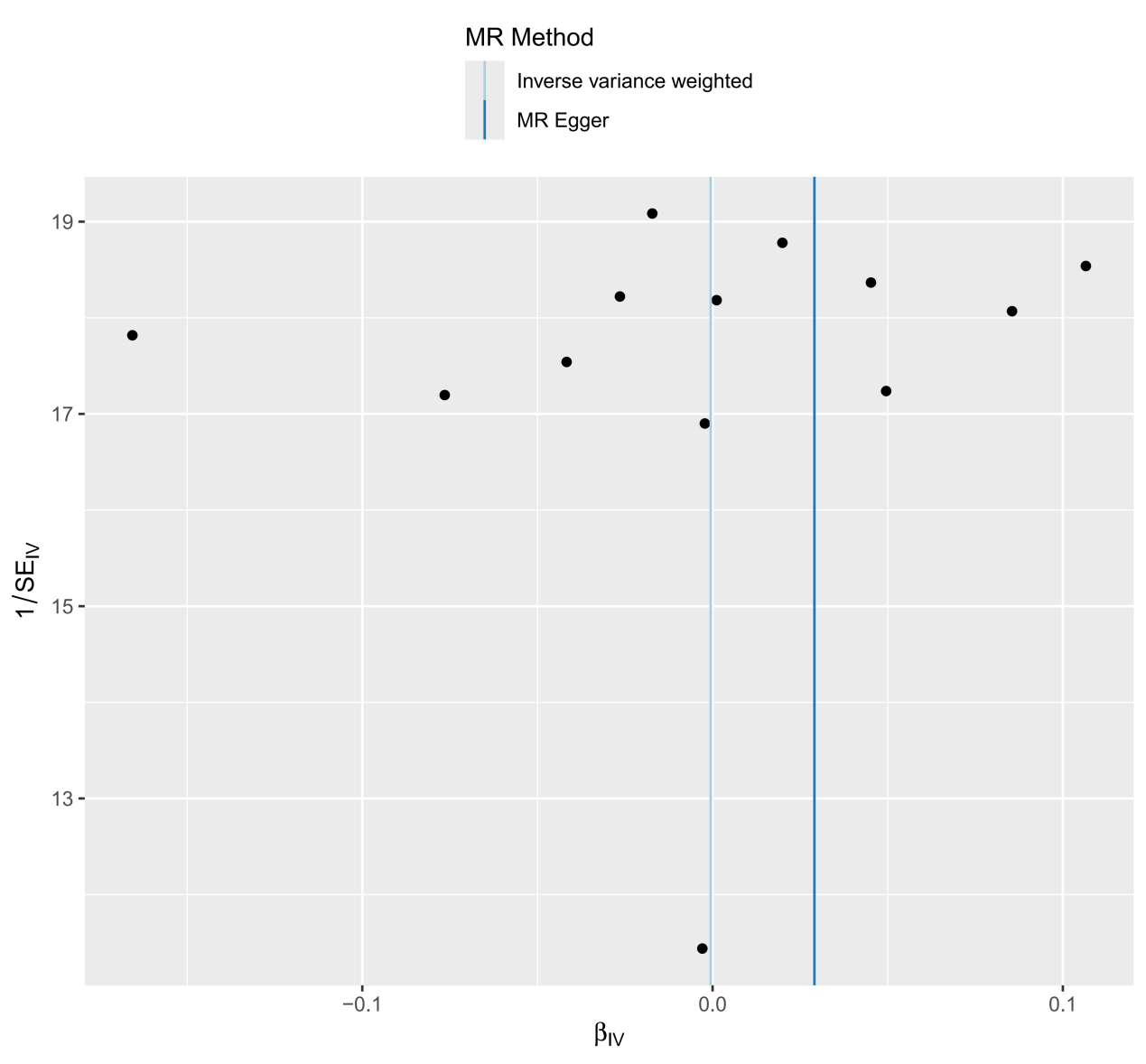


Supplementary Figure S3. Funnel plot of the Mendelian randomization analysis.


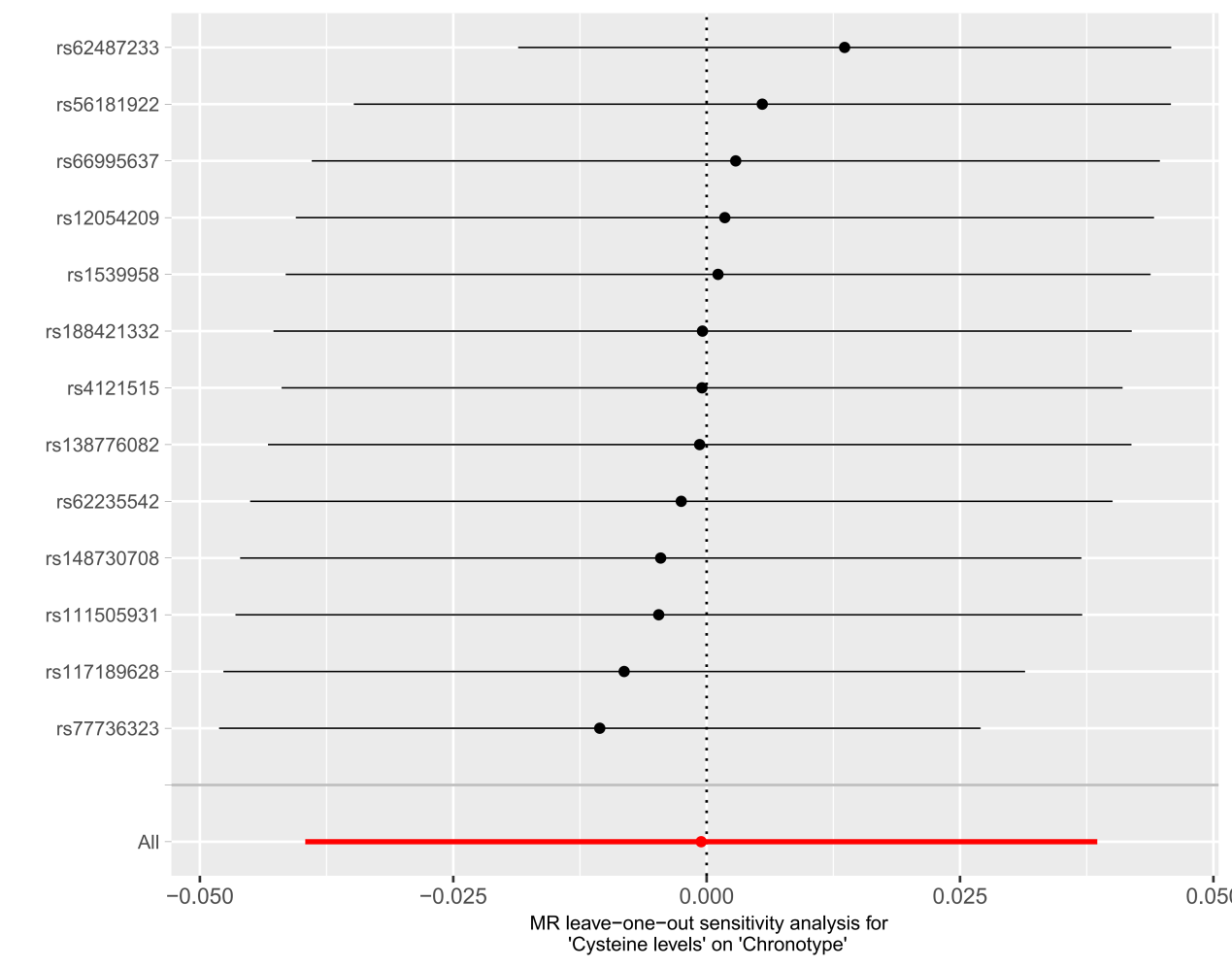


Supplementary Figure S4. Leave-one-out sensitivity analysis plot for the Mendelian randomization analysis.
