# Supplementary material for: Combining Mendelian Randomization and Experimental Validation to Reveal the Causal Relationship Between Hallux Valgus and Serum Metabolites and to Identify Their Therapeutic Targets and Relevant Components
Source: Food Sci Nutr. 2026 Jul 23;14(7):e72143. doi: 10.1002/fsn3.72143 (PMC13396431; doi:10.1002/fsn3.72143)
Supplement: Supplementary file 2 — Table S1: STROBE‐MR checklist. [file FSN3-14-e72143-s001.docx]

**Supplementary Table S1** STROBE-MR checklist.

**STROBE-MR checklist of recommended items to address in reports of Mendelian randomization studies**^1^ ^2^

| **Item No.** | **Section** | **Checklist item** | **Page No.** | **Relevant text from manuscript** |
| --- | --- | --- | --- | --- |
| 1 | **TITLE and ABSTRACT** | Indicate Mendelian randomization (MR) as the study’s design in the title and/or the abstract if that is a main purpose of the study | 1 and 2 to 3 | Line 1 to 4.Line 26 to line 62 |
|  | **INTRODUCTION** |  |  |  |
| 2 | **Background** | Explain the scientific background and rationale for the reported study. What is the exposure? Is a potential causal relationship between exposure and outcome plausible? Justify why MR is a helpful method to address the study question | 3 | Line 67 to 82 |
| 3 | **Objectives** | State specific objectives clearly, including pre-specified causal hypotheses (if any). State that MR is a method that, under specific assumptions, intends to estimate causal effects | 3 to 4 | Line 83 to 94 |
|  | **METHODS** |  |  |  |
| 4 | **Study design and data sources** | Present key elements of the study design early in the article. Consider including a table listing sources of data for all phases of the study. For each data source contributing to the analysis, describe the following: | 4 | 2.1 Data sources and methods of analysis |
|  | a) | Setting: Describe the study design and the underlying population, if possible. Describe the setting, locations, and relevant dates, including periods of recruitment, exposure, follow-up, and data collection, when available. | 4 | Line 96 to 114 |
|  | b) | Participants: Give the eligibility criteria, and the sources and methods of selection of participants. Report the sample size, and whether any power or sample size calculations were carried out prior to the main analysis | 4 to 5 | Line 96 to 114 |
|  | c) | Describe measurement, quality control and selection of genetic variants | 4 to 6 | Line 115 to 148 |
|  | d) | For each exposure, outcome, and other relevant variables, describe methods of assessment and diagnostic criteria for diseases | 6 to 7 | Line 149 to 179 |
|  | e) | Provide details of ethics committee approval and participant informed consent, if relevant | 16 | Line 458 to 463 |
| 5 | **Assumptions（假设）** | Explicitly state the three core IV assumptions for the main analysis (relevance, independence and exclusion restriction) as well assumptions for any additional or sensitivity analysis | 4 to 6 | Line 115 to 148 |
| 6 | **Statistical methods: main analysis** | Describe statistical methods and statistics used | 7 to 8 | Line 203 to 211 |
|  | a) | Describe how quantitative variables were handled in the analyses (i.e., scale, units, model) | 4 to 5 | Line 96 to 138 |
|  | b) | Describe how genetic variants were handled in the analyses and, if applicable, how their weights were selected | 4 to 6 | Line 96 to 138, line 143 to 148 |
|  | c) | Describe the MR estimator (e.g. two-stage least squares, Wald ratio) and related statistics. Detail the included covariates and, in case of two-sample MR, whether the same covariate set was used for adjustment in the two samples | 4 to 6 | Line 96 to 138, line 143 to 148 |
|  | d) | Explain how missing data were addressed | N/R | N/R |
|  | e) | If applicable, indicate how multiple testing was addressed | 7 | Line 197 to 200 |
| 7 | **Assessment of assumptions** | Describe any methods or prior knowledge used to assess the assumptions or justify their validity | 6 to 7 | Line 149 to 179 |
| 8 | **Sensitivity analyses and additional analyses** | Describe any sensitivity analyses or additional analyses performed (e.g. comparison of effect estimates from different approaches, independent replication, bias analytic techniques, validation of instruments, simulations) | 6 to 7 | Line 149 to 179 |
| 9 | **Software and pre-registration** |  |  |  |
|  | a) | Name statistical software and package(s), including version and settings used | 4 to 6 | Line 95 to 166 |
|  | b) | State whether the study protocol and details were pre-registered (as well as when and where) | 6 | Line 157 to 159 |
|  | **RESULTS** |  |  |  |
| 10 | **Descriptive data** |  |  |  |
|  | a) | Report the numbers of individuals at each stage of included studies and reasons for exclusion. Consider use of a flow diagram | 8 | Line 213 to 230 |
|  | b) | Report summary statistics for phenotypic exposure(s), outcome(s), and other relevant variables (e.g. means, SDs, proportions) | 8 | Line 213 to 230 |
|  | c) | If the data sources include meta-analyses of previous studies, provide the assessments of heterogeneity across these studies | N/A | The data source is GWAS aggregated data, not a meta-analysis. |
|  | d) | For two-sample MR:  i.  Provide justification of the similarity of the genetic variant-exposure associations between the exposure and outcome samples  ii.  Provide information on the number of individuals who overlap between the exposure and outcome studies | 8 to 9 | Line 213 to 264 |
| 11 | **Main results** |  |  |  |
|  | a) | Report the associations between genetic variant and exposure, and between genetic variant and outcome, preferably on an interpretable scale | 8 | Line 213 to 230 |
|  | b) | Report MR estimates of the relationship between exposure and outcome, and the measures of uncertainty from the MR analysis, on an interpretable scale, such as odds ratio or relative risk per SD difference | 8 to 9 | Line 213 to 264 |
|  | c) | If relevant, consider translating estimates of relative risk into absolute risk for a meaningful time period | 8 to 9 | Line 213 to 264 |
|  | d) | Consider plots to visualize results (e.g. forest plot, scatterplot of associations between genetic variants and outcome versus between genetic variants and exposure) | 8 to 9 | Line 213 to 264 |
| 12 | **Assessment of assumptions** |  |  |  |
|  | a) | Report the assessment of the validity of the assumptions | 9 | Line 256 to 264 |
|  | b) | Report any additional statistics (e.g., assessments of heterogeneity across genetic variants, such as *I^2^*, Q statistic or E-value) | 8 to 9 | Line 213 to 264 |
| 13 | **Sensitivity analyses and additional analyses** |  |  |  |
|  | a) | Report any sensitivity analyses to assess the robustness of the main results to violations of the assumptions | 8 to 9 | Line 205 to 215, line 236 to 247 |
|  | b) | Report results from other sensitivity analyses or additional analyses | 9 | Line 256 to 264 |
|  | c) | Report any assessment of direction of causal relationship (e.g., bidirectional MR) | 8 to 9 | Line 213 to 264 |
|  | d) | When relevant, report and compare with estimates from non-MR analyses | 9 | Line 256 to 264 |
|  | e) | Consider additional plots to visualize results (e.g., leave-one-out analyses) | - | Figure 1C-D and Figure 4C-D |
|  | **DISCUSSION** |  |  |  |
| 14 | **Key results** | Summarize key results with reference to study objectives | 11 to 12 | Line 309 to 329 |
| 15 | **Limitations** | Discuss limitations of the study, taking into account the validity of the IV assumptions, other sources of potential bias, and imprecision. Discuss both direction and magnitude of any potential bias and any efforts to address them | 11 to 12 and 14 | Line 309 to 329, line 403 to 407 |
| 16 | **Interpretation** |  |  |  |
|  | a) | Meaning: Give a cautious overall interpretation of results in the context of their limitations and in comparison with other studies | 11 to 14 | Line 309 to 396 |
|  | b) | Mechanism: Discuss underlying biological mechanisms that could drive a potential causal relationship between the investigated exposure and the outcome, and whether the gene-environment equivalence assumption is reasonable. Use causal language carefully, clarifying that IV estimates may provide causal effects only under certain assumptions | 11 to 14 | Line 309 to 396 |
|  | c) | Clinical relevance: Discuss whether the results have clinical or public policy relevance, and to what extent they inform effect sizes of possible interventions | 14 | Line 409 to 415 |
| 17 | **Generalizability** | Discuss the generalizability of the study results (a) to other populations, (b) across other exposure periods/timings, and (c) across other levels of exposure | 14 | Line 409 to 415 |
|  | **OTHER INFORMATION** |  |  |  |
| 18 | **Funding** | Describe sources of funding and the role of funders in the present study and, if applicable, sources of funding for the databases and original study or studies on which the present study is based | 16 | Line 411 to 445 |
| 19 | **Data and data sharing** | Provide the data used to perform all analyses or report where and how the data can be accessed, and reference these sources in the article. Provide the statistical code needed to reproduce the results in the article, or report whether the code is publicly accessible and if so, where | 16 | Line 454 to 456 |
| 20 | **Conflicts of Interest** | All authors should declare all potential conflicts of interest | 16 | Line 466 to 467 |

This checklist is copyrighted by the Equator Network under the Creative Commons Attribution 3.0 Unported (CC BY 3.0) license.

1. Skrivankova VW, Richmond RC, Woolf BAR, Yarmolinsky J, Davies NM, Swanson SA, et al. Strengthening the Reporting of Observational Studies in Epidemiology using Mendelian Randomization (STROBE-MR) Statement. JAMA. 2021;under review.

2. Skrivankova VW, Richmond RC, Woolf BAR, Davies NM, Swanson SA, VanderWeele TJ, et al. Strengthening the Reporting of Observational Studies in Epidemiology using Mendelian Randomisation (STROBE-MR): Explanation and Elaboration. BMJ. 2021;375:n2233.
