# Supplementary material for: Combining Mendelian Randomization and Experimental Validation to Reveal the Causal Relationship Between Hallux Valgus and Serum Metabolites and to Identify Their Therapeutic Targets and Relevant Components
Source: Food Sci Nutr. 2026 Jul 23;14(7):e72143. doi: 10.1002/fsn3.72143 (PMC13396431; doi:10.1002/fsn3.72143)
Supplement: Supplementary file 3 — Table S2: Negative control analysis results of the causal association between Cysteine and Chronotype using Mendelian randomization. [file FSN3-14-e72143-s002.docx]

**Supplementary Table S2** Negative control analysis results of the causal association between Cysteine and Chronotype using Mendelian randomization.

| id.exposure | id.outcome | outcome | exposure | method | nsnp | b | se | pval | lo_ci | up_ci | or | or_lci95 | or_uci95 |
| --- | --- | --- | --- | --- | --- | --- | --- | --- | --- | --- | --- | --- | --- |
| GCST90200439 | ieu-b-4862 | Chronotype | Cysteine levels | MR Egger | 13 | 0.0290664202472672 | 0.0428906207016593 | 0.51197235691517 | -0.054999196 | 0.113132036822519 | 1.02949297138202 | 0.946485908617416 | 1.11977977535151 |
| GCST90200439 | ieu-b-4862 | Chronotype | Cysteine levels | Weighted median | 13 | -0.000785566 | 0.0226828259240538 | 0.972372669445862 | -0.045243905 | 0.0436727725329814 | 0.999214742198243 | 0.9557643376156 | 1.04464046390049 |
| GCST90200439 | ieu-b-4862 | Chronotype | Cysteine levels | Inverse variance weighted | 13 | -0.000539264 | 0.0199278835931789 | 0.978411242379813 | -0.039597916 | 0.038519387420504 | 0.999460880954798 | 0.961175834635263 | 1.03927087694418 |
| GCST90200439 | ieu-b-4862 | Chronotype | Cysteine levels | Simple mode | 13 | -0.003805821 | 0.0382553577828019 | 0.922396123803132 | -0.078786322 | 0.0711746801383252 | 0.996201411842525 | 0.924237392202399 | 1.07376877556552 |
| GCST90200439 | ieu-b-4862 | Chronotype | Cysteine levels | Weighted mode | 13 | -0.003805821 | 0.0384749507073963 | 0.92283747568974 | -0.079216725 | 0.0716050822705302 | 0.996201411842525 | 0.923839684051489 | 1.07423102740597 |
